# Indoleamine-2,3-Dioxygenase Mediates Emotional Deficits by the Kynurenine/Tryptophan Pathway in the Ethanol Addiction/Withdrawal Mouse Model

**DOI:** 10.3389/fncel.2020.00011

**Published:** 2020-02-11

**Authors:** Xi Jiang, Qian Lin, Lexing Xu, Ziwei Chen, Qizhi Yan, Lei Chen, Xuefeng Yu

**Affiliations:** ^1^Department of Pharmacy, Zhejiang Pharmaceutical College, Ningbo, China; ^2^Mingzhou Hospital, Zhejiang University, Hangzhou, China; ^3^Department of Pharmacology & Toxicology, University of Louisville, Louisville, KY, United States; ^4^Shaoxing People’s Hospital, Zhejiang University School of Medicine, Shaoxing, China

**Keywords:** ethanol addiction, indoleamine 2, 3-dioxygenase, depression, anxiety, memory

## Abstract

**Objective:**

Our study was designed to investigate whether the indoleamine-2,3-dioxygenase (IDO)-mediated kynurenine/tryptophan (KYN/TRP) pathway participates in the development of emotional deficits from ethanol addiction/withdrawal mice.

**Methods:**

The expression of proinflammatory factors, including tumor necrosis factor α (TNF-α), interleukin-1β (IL-1β), and interleukin-6 (IL-6), was tested by enzyme-linked immunosorbent assay (ELISA). The IDO levels in the hippocampus, cerebral cortex, and amygdala were measured by polymerase chain reaction (PCR) and western blot, and the neurotransmitters were tested by high performance liquid chromatography (HPLC). Emotional deficits of mice were evaluated by behavioral tests.

**Results:**

Expression levels of inflammatory factors (TNF-α, IL-1β, and IL-6) were increased in mice after 4 weeks of alcohol exposure. As for indoleamine 2,3-dioxygenase (IDO) expression, only the subtype IDO1 was found to increase at both mRNA level and protein level in all the tested brain regions of ethanol addiction/withdrawal mice. In behavioral tests, mice exposed to alcohol showed gradually declined memory function accompanied by anxiety-like and depressive-like behaviors. Meanwhile, increased expression of KYN, decreased expression of 5-HT, and abnormal expression of 3-HK and KA were found in the hippocampus, cerebral cortex, and amygdala of ethanol addiction/withdrawal mice. Interestingly, the IDO1 inhibitor, 1-methyl-L-tryptophan (1-MT), reversed all above alterations induced by ethanol in mice.

**Conclusion:**

Our results suggested that the TRP/KYN pathway, medicated by IDO1, in the hippocampus, cerebral cortex, and amygdala, plays an important role in the development of emotional deficits caused by ethanol addiction and withdrawal.

## Introduction

Alcohol is one of the most popular drinks worldwide. It has been demonstrated that moderate drinking is beneficial to health. For example, it can reduce cardiovascular disease mortality and increase life expectancy (Guzmán et al., 2004; Orio et al., 2019). However, alcohol is also a widely used psychoactive drug which causes many public health problems in our society. For instance, excessive drinking of alcohol results in multiple medical complications and significantly increases the risk of central nerves system diseases.

A growing amount of evidence suggests that alcohol addiction-induced physiological lesions and brain nerve injuries were strongly associated with neuroinflammation (Obernier et al., 2002; Tilg et al., 2016). Patients with alcohol addiction showed not only emotional deficits, such as depression, anxiety, and cognitive impairment, but also significantly increased expression of proinflammatory cytokines in the serum and brain. The behavioral impairments would deteriorate during the process of alcohol withdrawal if the patients received no treatment (Maier, 2001; Pascual et al., 2011). However, the crosstalk of brain-derived neuroinflammation and behavioral deficits in the process of alcohol addiction and withdrawal have not been clearly studied.

As a catalyst, the enzyme IDO takes an active role in the formation of KYN from TRP during the neuroregulatory metabolism and is markedly activated by proinflammatory cytokines. Many recent studies demonstrated that the IDO-mediated KYN/TRP pathway plays an important role in the development of neurological damages (André et al., 2008; O’Connor et al., 2009a, b; Naseem and Bano, 2018). 5-hydroxytryptamine (5-HT) is a kind of neurotransmitter and is strongly related to depression, anxiety, and memory function. In normal organisms, the main metabolite of TRP is 5-HT, which could help to improve emotion (Ganong and Cotman, 1986). Under inflammatory conditions, The TRP metabolic pathway will be changed because of the activated IDO. Most of TRP would be metabolized to KYN and would then be further metabolized to kynurenic acid (KA), which is a neurotoxic metabolite (Stone and Perkins, 1981; Walker et al., 2013). Therefore, the activated IDO induces abnormal TRP metabolism, causing the development of neurotoxicity and deficiency of 5-HT in the brain (Dantzer et al., 2008; Parrott and O’Connor, 2015).

The IDO-mediated KYN pathway of TRP metabolism in alcohol addiction/withdrawal disease has rarely been investigated. Our present study has attempted to explore the potential role of the IDO-mediated KYN/TRP pathway in the development of behavioral impairments in an alcohol addiction/withdrawal mouse model.

## Materials and Methods

### Animals

Male ICR mice (4–6 weeks old) weighing 20–22 g were provided by the Animal Experimental Center of Wenzhou Medical University. The mice were housed and acclimated to a colony room with controlled ambient temperature (22 ± 1°C), humidity (50 ± 10%) and a 12-h light/12-h dark cycle. All the experimental procedures were approved by the Animal Care and Use Committee of Wenzhou Medical University. The mice experiments were designed as an 8-week study, and mice were randomly divided into five groups (0, 2, 4, 6, and 8 weeks). Each group contained five subgroups: control, ethanol, and ethanol supplemented with different doses (0.1, 1, and 3 mg/kg body weight) of 1-MT. As performing multiple tests on the same mouse may cause inaccurate results, each subgroup was divided into two sets (*n* = 8 per set). Animals in one set were used for a tail suspension test, forced swimming test, marble-burying test, and elevated plus maze test to study anxiety and depressive-like behavior. Animals in another set were used for a Morris water maze test and locomotor activity test. All the mice received an ethanol preference test.

### Induction of Alcohol Addiction

The animal model of chronic alcohol addiction was constructed based on the method used by Lowe et al. (2018) with minor modification. The concentrations of ethanol at the 1st, 2nd, 3rd, and 4th week were 5, 10, 20, and 35%, respectively. Ethanol concentration was kept at 35% from the 5th to 8th week. In each week, mice received no ethanol for 24 h before behavioral tests. After behavior tests, mice restarted to drink ethanol. This cycle was designed to mimic the process of patient’s withdrawal and re-drinking in clinic. The ethanol solution was used as the unique source of drinking water.

### 1-MT Treatment

**FIGURE 1:**
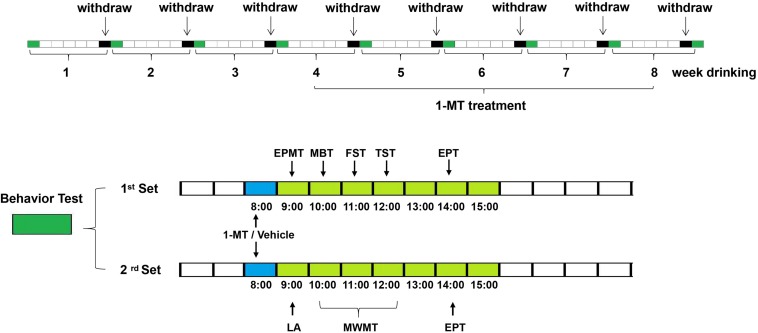
Scheme of the experimental design. Mice received ethanol drinking for 8 weeks and received the first administration of 1-MT (0.1, 1, and 3 mg/kg, i.p.) or vehicle after 4 weeks of drinking; behavior tests were done at 0, 2, 4, 6, and 8 weeks after drinking. Each group contains five subgroups as follows: control, ethanol, and ethanol supplemented with different doses (0.1, 1, and 3 mg/kg body weight) of 1-MT. Each subgroup was divided into two sets (*n* = 8 per set). The mice in the 1st set received elevated plus maze test at 9:00, marble-burying test at 10:00, forced swimming test at 11:00, tail suspension test at 12:00, and ethanol preference test at 14:00. The mice in the 2nd set received a locomotor activity test at 9:00, Morris water maze test at 10:00–12:00, and ethanol preference test at 14:00.

Mice received 1-MT (Sigma-Aldrich) at week 5 (4 weeks after ethanol exposure). 1-MT was dissolved in physiological saline to prepare different doses (0.1, 1, and 3 mg/kg) of 1-MT solution. Each mouse received 1-MT solution through intraperitoneal injection. Control and model mice were injected with equal volumes of normal saline. The experimental design was summarized in Figure 1. The locomotor activity test was performed first and was then followed by other behavioral tests. After behavioral tests, mice in each group were sacrificed at 0, 2, 4, 6, and 8 weeks, respectively. The hippocampus, cerebral cortex, and amygdala were rapidly dissected out and stored at −80°C.

### Behavioral Training

Each mouse received behavioral tests after ethanol withdrawal at 0, 2, 4, 6, and 8 weeks. The behavioral tests were conducted between 9:00 am and 3:00 pm of on monday in each week.

#### Locomotor Activity

Each mouse was placed in the testing chamber (45 cm × 45 cm × 45 cm), which was linked to a behavior testing instrument. Mice were placed in the chambers with their paws contacting or disconnecting the active bars, which produced random configurations that were converted into pulses. The pulses, which were proportional to the locomotor activity of the mice, were automatically recorded as the cumulative total counts of motor activity. The experiment lasted for 10 min. The chamber was wiped with alcohol when a test was completed before the next one (Yu et al., 2016).

#### Ethanol Preference Test

Mice were fed with ethanol solution (5% ethanol) and ordinary drinking water for 24 h before testing. Afterward, each mouse received these two solutions for 1 h, and the result was calculated as the volume ratio of ethanol solution/total intake (ethanol intake + water intake).

#### Tail Suspension Test

The tail suspension test (TST) was performed in accordance to the previous research (Jiang et al., 2017a). In brief, the mouse was suspended 50 cm above the floor using adhesive tape, placed approximately 1 cm from the tip of the tail. The experiment lasted for 6 min. The immobility time during the last 4 min was calculated.

#### Forced Swimming Test

The forced swimming test (FST) was performed according to previous research (Jiang et al., 2017b). Each mouse was placed in the glass cylinder (height: 30 cm; diameter: 10 cm; containing 20 cm depth of water with a temperature of 24 ± 1°C) for 6 min. A mouse was judged to be immobile when it stopped struggling and remained floating motionless in the water. The experiment lasted for 6 min, and immobility time was recorded during the last 4 min.

#### Marble-Burying Test

In brief, each mouse was placed in a polypropylene cage (45 cm × 45 cm × 45 cm) with nine clean glass marbles (diameter: 2.3 cm), which were evenly spaced on sawdust at depth of 5 cm. The number of marbles buried by each animal was calculated in the experimental period of 10 min (Jiang et al., 2016).

#### Elevated Plus Maze Test

This experiment was conducted according to the previous research (Jiang et al., 2016). Each mouse was firstly placed on the central area of an elevated plus maze. The number of entries in close arm, the time spent in the close and open arms were recorded by tracking software (JZZ98, Wenzhou Medical College) in the experimental period of 10 min.

#### Morris Water Maze Test

For Morris water maze test, the apparatus consisted of a circular plastic pool (diameter: 95 cm × high: 25 cm) located in a well-illuminated room with external cues visible from the inside of the pool and filled with opaque water (24 ± 1°C). The experimental pool was divided into four areas. A hidden circular platform (diameter: 8.5 cm × high: 15.5 cm) was placed in one of the four areas. Each mouse firstly received the acquisition training, which contained their trials. In each trial, a mouse was put in the water at four different directions (east, south, west, and north) (60 s training in each direction). If the mice did not find the platform within the training period of 60 s, we helped the mouse stay on the platform for 5 s. Twenty-four hours later, we removed the platform and placed the animal into water in the east direction of the pool. The latency to find the previous platform and number of crossings over the target quadrant were recorded by tracking software (JZZ98, Wenzhou Medical College) in an experimental period of 60 s (Xu et al., 2015).

### Biochemical Analysis

Expressions of IDO subtypes in brain tissues were detected by PCR and immuno-blot. Proinflammatory cytokine expressions were detected via ELISA assay. Levels of TRP and TRP metabolites were tested by HPLC. The amount of tissue in the brain region in one mouse was limited for all the biochemical analyses, so tissues of animals in one group were combined and homogenized and then divided into several parts for different biochemical detections.

#### High Performance Liquid Chromatography (HPLC)

The method for HPLC was based on previous research (O’Connor et al., 2009a). Each sample was added with 50 μL lysate, and supernatants were extracted and centrifuged at 12,000 rpm for 5 min. The mobile phase for KYN and TRP was 75 mM NaH2PO4, 25 μM EDTA, and 100 μL/L triethylamine diluted in acetonitrile/water (6:94 v/v) solution (pH = 4.6). The mobile phase for 5-HT, 5-HIAA, 3-HK, QA, and KA was 75 mM NaH2PO4, 25 μM EDTA, 0.45 mM octanesulfonic acid, and 100 μL/L triethylamine diluted in acetonitrile/water (6:94 v/v) solution (pH = 3.0).

#### Quantitative Real-Time RT-PCR

Tissue samples were prepared according to the RNA kit (Bio-Rad. Labs). The primer sequences of target RNAs are shown in Table 1. RNA amplification cycle was based on previous research (Jiang et al., 2017b).

**TABLE 1:** Primer sequences of IDOs.

**Target**	**Forward (5′–3′)**	**Reverse (5′–3′)**
IDO1	AGAAGTGGGCTTTGCTCTGC	TGGCAAGACCTTACGGACATCTC
IDO2	AAGCTTATGGAGCCTCAAAGTCAGAGC	CTCGAGCTAAGCACCAGGACACAGG
TDO	TGGGAACTAGATTCTGTTCG	TCGCTGCTGAAGTAAGAGCT
β-actin	TGGAATCCTGTGGCATCCATGAAAC	AAAACGCAGCTCAGTAACAGTCCG

*TDO, tryptophan-2,3-dioxygenase.*

#### Immuno-Blot Analysis

A BCA kit (Thermo Scientific, Shanghai, China) was used to determine protein concentration of each sample. Each band contained a total protein of 40 μg. After electrophoresis and membrane transferring, blots were blocked with milk for 2 h and then incubated with primary antibodies (anti-IDO, 1:1000; anti-IDO2, 1:1000; anti-β-actin, 1:1000 from Santa Cruz biotechnology, Shanghai, China). After washing and incubating with secondary antibodies, blots were imaged by fluorescence scanner, and data were analyzed.

#### Enzyme-Linked Immunosorbent Assay (ELISA)

The experimental operation was based on ELISA kit (Thermo Scientific, Shanghai, China). The OD values of tumor necrosis factor α (TNF-α), interleukin-1β (IL-1β), and interleukin-6 (IL-6) were detected at 450 nm wavelength.

### Statistical Analysis

A two-way analysis of variance (two-way ANOVA) and multi-way ANOVA were used to analyze the differences between data. For the two-way ANOVA, group factors were the procedures (control or ethanol administration) and the time was considered as the within-group factor. For the multi-way ANOVA, group factors were the procedure (control or ethanol administration) and the treatment (saline vs. 1-MT treatment), and the within-group factor was time. A Duncan test was used for *post hoc* analysis. All data are expressed as mean ±SD. *P*-value less than 0.05 was considered as a statistical difference.

## Results

### Expression of Proinflammatory Cytokines in the Mice Exposed to Ethanol

To investigate the time course and proinflammatory cytokine expressions in response to ethanol exposure, levels of IL-1β, TNF-α, and IL-6 in the hippocampus, cerebral cortex, and amygdala were tested at 0, 2, and 4 weeks of alcohol exposure.

**FIGURE 2:**
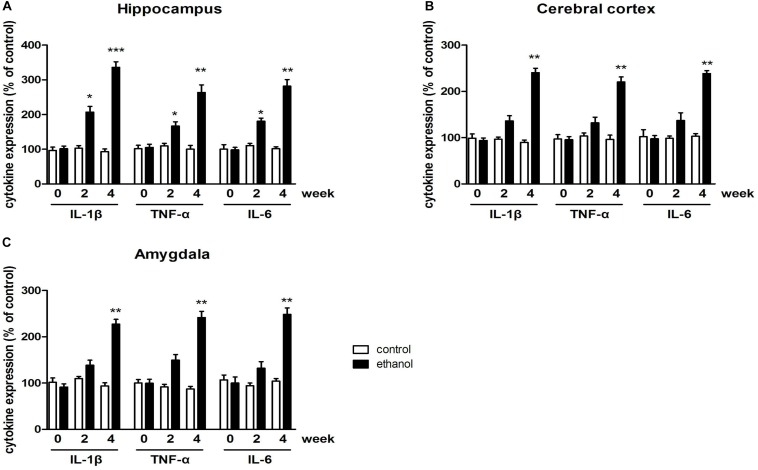
Levels of TNF-α, IL-1β, and IL-6 in the hippocampus **(A)**, cerebral cortex **(B)**, amygdala **(C)** in drinking mice at 0, 2, and 4 weeks. *n* = 8 per group, and data were assessed by two-way ANOVA followed by a Duncan test. *p < 0.05, **p < 0.01, and ***p < 0.001 compared with control group.

Data of proinflammatory cytokine expressions were summarized in Figure 2. Significant increases of TNF-α, IL-1β, and IL-6 expressions in the hippocampus of mice were observed after 4 weeks of drinking (*p* < 0.001, *p* < 0.01, Figure 2A). The increases of TNF-α, IL-1β, and IL-6 expressions were also found in the cerebral cortex and amygdala (*p* < 0.01 in all case, Figures 2B,C). These data indicated that neuroinflammation was triggered by alcohol in the mice.

#### Expression of IDO in the Mice of Ethanol Addiction

**FIGURE 3:**
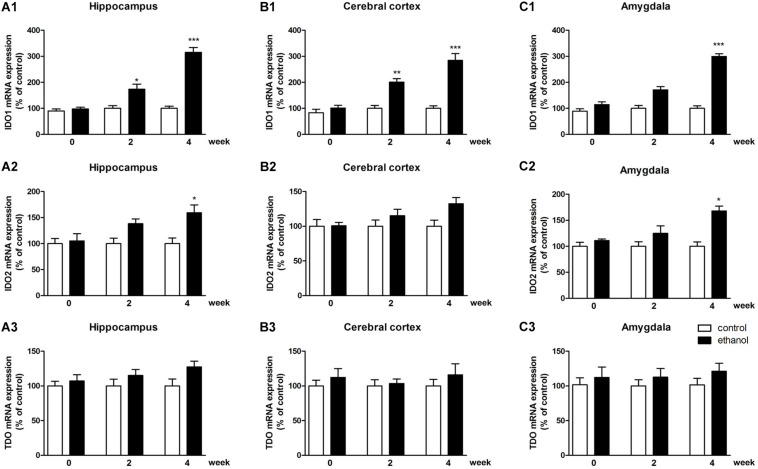
mRNA expressions of IDO1 **(A1–C1)**, IDO2 **(A2–C2)** and TDO **(A3–C3)** in the hippocampus, cerebral cortex, and amygdala in drinking mice at 0, 2, and 4 weeks. *n* = 8 per group, and data were assessed by two-way ANOVA followed by a Duncan test. *p < 0.05, **p < 0.01, and ***p < 0.001 compared with control group.

To identify whether any subtype of IDO was associated with ethanol addiction, the expressions of IDO1, IDO2, and TDO were tested by PCR and western blot. As shown in Figure 3, significant increases of IDO1 mRNA levels were observed in the hippocampus, cerebral cortex, and amygdala of mice after 4 weeks of drinking (*p* < 0.001, Figures 3A1–C1) as compared with the control mice group. For IDO2, 4 weeks of drinking increased the IDO2 mRNA expressions in the hippocampus and amygdala (*p* < 0.05, Figures 3A2–C2) but not in cerebral cortex of mice. Moreover, no significant difference of TDO mRNA expression was observed after drinking in all these brain regions (Figures 3A3–C3).

**FIGURE 4:**
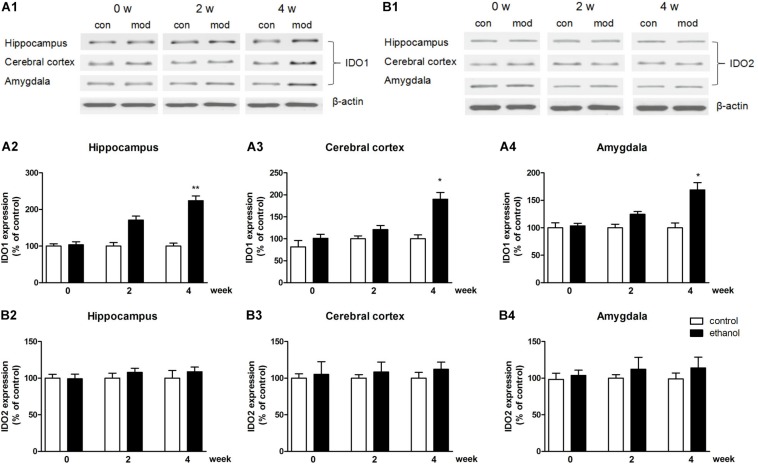
Protein expressions of IDO1 **(A1–A4)** and IDO2 **(B1–B4)** in the hippocampus, cerebral cortex, and amygdala in drinking mice at 0, 2, and 4 weeks. *n* = 8 per group, and data were assessed by two-way ANOVA followed by a Duncan test. *p < 0.05 and **p < 0.01 compared with control group. Con, control group; mod, model group (drinking group).

In terms of protein level, the expression of IDO1 was increased in the hippocampus, cerebral cortex, and amygdala of mice (*p* < 0.01, *p* < 0.05, Figures 4A1–A4), while the protein expression changes of IDO2 and TDO could not be observed in all these brain regions (Figures 4B1–B4 and Supplementary Figure S1: data of TDO protein expression).

### Locomotor Activity of Mice

**FIGURE 5:**
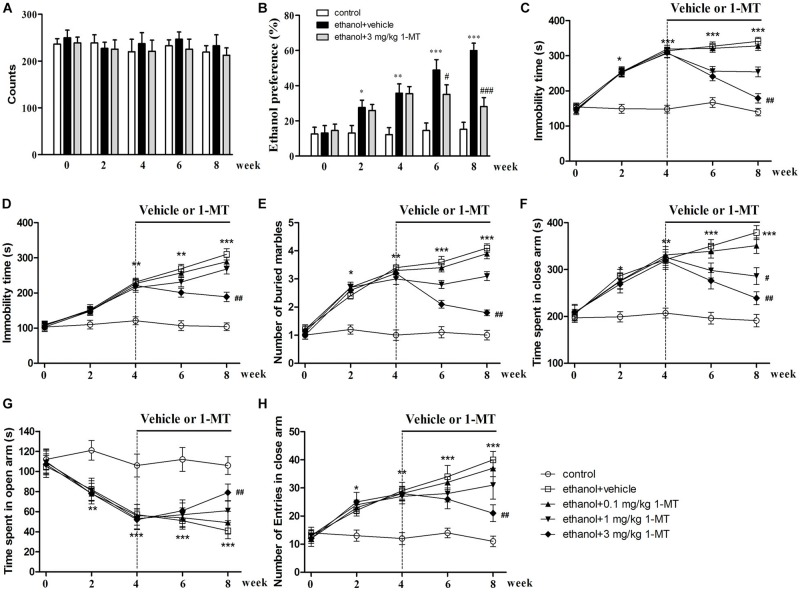
Effects of 1-MT on locomotor activity **(A)**, ethanol preference test **(B)**, forced swimming test **(C)**, tail suspension test **(D)**, marble-burying test **(E)**, and elevated plus maze test **(F–H)** in drinking mice at week 0–8. *n* = 8 per group, and data were assessed by multi-way ANOVA followed by a Duncan test. *p < 0.05, **p < 0.01, and ***p < 0.001 compared with control group; #*p* < 0.05, ##*p* < 0.01, and ###*p* < 0.001 compared with drinking group.

The behavior test of locomotor activity was performed to eliminate the excitatory or inhibitory effects of 1-MT. As shown in Figure 5A, the locomotor activities of mice presented no significant difference between control group and ethanol drinking group at each time point. Meanwhile, 1-MT did not influence locomotor activity of control mice and mice exposed to alcohol [Supplementary Figure S2: data of 1-MT (0.1 and 1 mg/kg), Supplementary Figure S6: data of 1-MT (3 mg/kg)].

### Effects of 1-MT on the Developments of Ethanol Addiction and Ethanol Addiction-Induced Depressive-Like Behaviors in Mice

To explore the role of IDO1 in ethanol addiction and depressive-like behaviors of mice exposed to alcohol, IDO1 inhibitor 1-MT was employed, and each mouse received ethanol preference, force swimming, and tail suspension tests. The mice exposed to alcohol showed a remarkable preference for ethanol after 8 weeks of drinking (*p* < 0.001, Figure 5B). With 1-MT treatment for 4 weeks (from the 4th to 8th week), the obvious ethanol preference of mice was markedly inhibited at the 8th week (*p* < 0.001, Figure 5B). In the forced swimming test, ethanol drinking induced a significant increase of immobility time as compared to the control mice at the 4th week, and this increase lasted till the 8th week (*p* < 0.001, Figure 5C). 1-MT treatment at 3 mg/kg for 4 weeks, however, significantly decreased the immobility time in mice exposed to alcohol (*p* < 0.01, Figure 5C).

The protective effects of 1-MT on depressive-like behaviors of mice were further demonstrated in a tail suspension test. A significant increase of immobility time in the tail suspension test was observed in mice after 4 weeks of drinking (*p* < 0.01, Figure 5D), and this depressive behavior became more pronounced after 8 weeks of drinking. This phenomenon was significantly reversed by chronic treatment with 1-MT at 3 mg/kg for 4 weeks (from the 4th to 8th week, *p* < 0.01, Figure 5D). Furthermore, 1-MT treatment at 3 mg/kg did not affect the immobility time of FST and TST in control mice (Supplementary Figure S6). These data indicated that IDO1 was involved in drinking-induced addiction and depressive-like behavior in the mice.

### Effects of 1-MT on the Development of Ethanol Addiction-Induced Anxiety-Like Behaviors in Mice

To explore the role of IDO1 in anxiety-like behaviors in mice exposed to alcohol, each mouse received a marble-burying test and elevated plus maze test. For the marble-burying test, 4 weeks of drinking increased the number of buried marbles when compared with control group (*p* < 0.01, Figure 5E). Chronic administration with 1-MT at 3 mg/kg for 4 weeks (from the 4th to 8th week) decreased the number of buried marbles in mice exposed to alcohol (*p* < 0.01).

In the elevated plus maze test, 4 weeks of ethanol drinking obviously increased the time in the closed arms and decreased the exploration time in the open arms of mice, and these abnormal behaviors became more obvious at the 8th week (*p* < 0.001 in all cases, Figures 5F,G). Correspondingly, drinking also increased the number of entries into the closed arms at the 8th week (*p* < 0.001, Figure 5H). However, 3 mg/kg 1-MT treatment for 4 weeks prevented these anxiety-like behaviors (*p* < 0.01 in all cases). Furthermore, no significant differences were found between 3 mg/kg 1-MT treated control mice and control mice (Supplementary Figure S6). These results indicated that IDO1 was involved in the development of drinking-induced anxiety-like behaviors.

### Effects of 1-MT on the Development of Ethanol Addiction-Induced Memory Impairment in Mice

**FIGURE 6:**
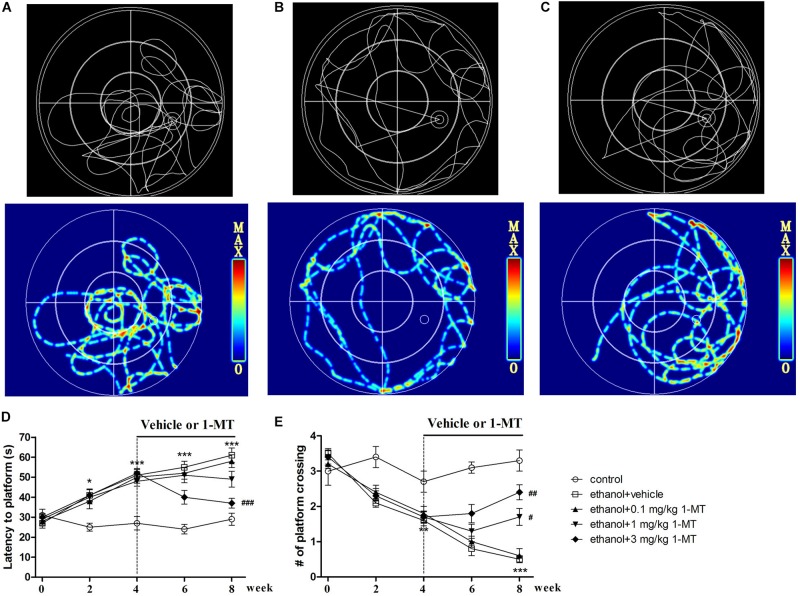
Effects of 1-MT on drinking-induced memory deficits in mice. Animal trajectory of control, model, and 3 mg/kg 1-MT groups are summarized in **(A–C)**. The second row is the hotspots image of the first row. Latency to platform **(D)** and number of platform crossing **(E)** were tested at different weeks (0, 2, 4, 6, and 8 week). *n* = 8 per group, data were assessed by multi-way ANOVA followed by a Duncan test. *p < 0.05, **p < 0.01, and ***p < 0.001 compared with control group; #*p* < 0.05, ##*p* < 0.01, and ###*p* < 0.001 compared with drinking group.

To disclose the role of IDO1 in memory deficit in mice exposed to alcohol, each mouse received a water maze test. The trajectories of the water maze test showed that control and 1-MT-treated mice concentrated more on the target area (Figures 6A–C). We found that time to find the platform was longer (*p* < 0.001, Figure 6D), and the number of passes through the platform was less (*p* < 0.001, Figure 6E), in mice exposed to alcohol in comparison with control mice after 8 weeks of drinking. However, 3 mg/kg 1-MT treatment for 4 weeks (from 4th to 8th week) significantly ameliorated these symptoms (*p* < 0.001 for latency to platform and *p* < 0.01 for platform crossing, at 8th week). The results indicated that IDO1 was involved in the development of memory impairment in mice exposed to alcohol.

### Effects of 1-MT on KYN/TRP Pathway in Mice Exposed to Alcohol

**FIGURE 7:**
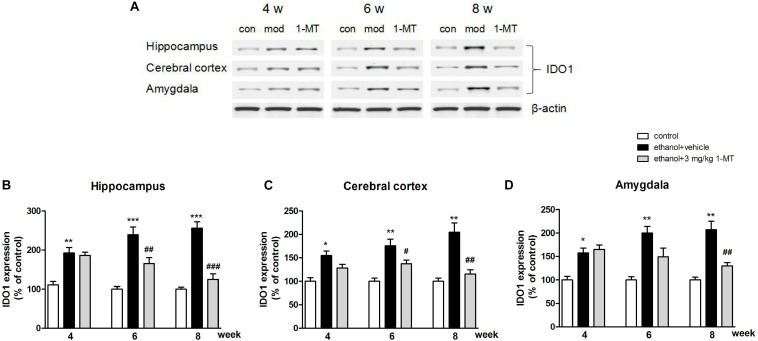
1-MT treatment decreased IDO1 expression in the hippocampus **(B)**, cerebral cortex **(C)**, and amygdala **(D)** in drinking mice at 4–8 weeks. Blots were summarized in **(A)**. *n* = 8 per group, and data were assessed by multi-way ANOVA followed by Duncan test. *p < 0.05, **p < 0.01, and ***p < 0.001 compared with control group; #*p* < 0.05, ##*p* < 0.01, and ###*p* < 0.001 compared with drinking group. Con, control group, mod, model group (drinking group).

Brain IDO1 level was shown to be increased in mice after ethanol exposure as mentioned above, while 1-MT (3 mg/kg) treatment for 4 weeks decreased the increased expressions of IDO1 in the hippocampus, cerebral cortex, and amygdala [*p* < 0.001, *p* < 0.01, and *p* < 0.01, respectively, Figure 7 and Supplementary Figure S3: data of treatment with 1-MT at low doses (0.1 and 1 mg/kg), Supplementary Figure S7: data of treatment with 1-MT at 3 mg/kg in control mice].

**FIGURE 8:**
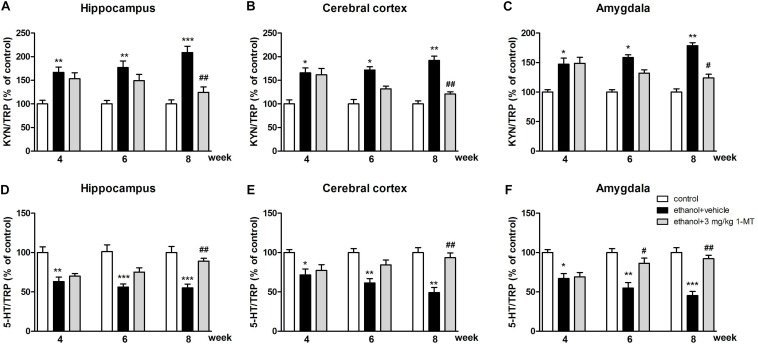
Effects of 1-MT on KYN/TRP ratio **(A–C)** and 5-HT/TRP ratio **(D–F)** in the hippocampus, cerebral cortex, and amygdala in drinking mice. *n* = 8 per group, and data were assessed by multi-way ANOVA followed by a Duncan test. *p < 0.05, **p < 0.01 and ***p < 0.001 compared with control group; #*p* < 0.05 and ##*p* < 0.01 compared with drinking group.

In addition, the increased KYN/TRP ratios and the decreased 5-HT/TRP ratios were observed in the hippocampus, cerebral cortex, and amygdala of mice exposed to alcohol (*p* < 0.001 for hippocampus, *p* < 0.01 for cerebral cortex and amygdala, Figure 8) at 8th week. Interestingly, all these changes were markedly reversed by 3 mg/kg 1-MT treatment [Figure 8, and Supplementary Figure S4: data of treatment with 1-MT at low doses (0.1 and 1 mg/kg)].

**FIGURE 9:**
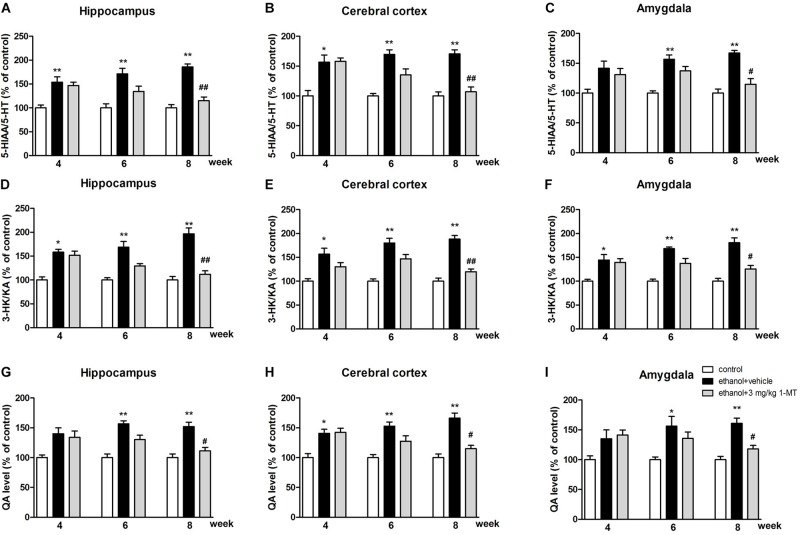
Effects of 1-MT on 5-HIAA/5-HT ratio **(A–C)**, 3-HK/KA ratio **(D–F)** and QA level **(G–I)** in the hippocampus, cerebral cortex, and amygdala of drinking mice. *n* = 8 per group, and data were assessed by multi-way ANOVA followed by a Duncan test. *p < 0.05 and **p < 0.01 compared with control group; #*p* < 0.05 and ##*p* < 0.01 compared with drinking group.

Furthermore, our data indicated that both the 5-HIAA/5-HT ratio and 3-HK/KA ratio were increased after 8 weeks of drinking in each brain region (*p* < 0.01 for the hippocampus, Figures 9A,D; *p* < 0.01 for the cerebral cortex and amygdala, Figures 9B,C,E,F). Moreover, a high expression of QA was also observed in the hippocampus, cerebral cortex, and amygdala (*p* < 0.01 in all cases, Figures 9G–I). Consistent with the upstream factors, the changes of all these neurotransmitters were reversed by 3 mg/kg 1-MT treatment for 4 weeks [Figure 9, and Supplementary Figure S5: data of treatment with 1-MT at low doses (0.1 and 1 mg/kg)]. The actual values of KYN, TRP, 5-HT, 5-HIAA, 3HK, and KA that contributed to the ratios are summarized in Supplementary Tables S1–S3. Furthermore, 3 mg/kg 1-MT treatment did not affect levels of KYN, TRP, 5-HIAA,5-HT, 3-HK, KA, and QA in control mice (Supplementary Table S4). The results indicated that the KYN/TRP pathway was activated upon the stimulation of alcohol, and this activation was mediated by IDO1.

## Discussion

Chronic drinking is a widely used animal model for studying ethanol addiction (Bertola et al., 2013; Sanchez-Alavez et al., 2018), but it takes a long time to establish the model, and it is not easy to generate the withdrawal symptoms. Therefore, a traditional chronic drinking model could not well simulate the clinical addiction/withdrawal process. In the present study, we added the withdrawal period every week to construct the process of ethanol withdrawal and re-drinking in mice. The ethanol addiction/withdrawal mice showed obvious depressive- and anxiety-like symptoms and memory impairment at the 4th week. This modified animal model is closer to a real clinical setting and takes a shorter time to model.

In our study, depressive-like and anxiety-like behaviors as well as memory deficits were found in ethanol addiction/withdrawal mice. It is in line with the results of previous researches (Crews et al., 2006; Tajuddin et al., 2014). Simultaneously, we found that proinflammatory cytokines were overexpressed in the hippocampus, cerebral cortex, and amygdala of mice after alcohol drinking. These results indicated that the development of emotional and memory deficits was most likely mediated by the activation of neuroinflammation in these brain regions, as suggested by the study reported by Chastain et al. (Chastain and Sarkar, 2014).

The three subtypes of IDO, IDO1, IDO2, and TDO, could be activated by many proinflammatory cytokines, such as TNF-α, IL-1β, and IL-6 (Sublette and Postolache, 2012; Naseem and Bano, 2018). Previous study reported that increased expression of inflammatory mediators caused by an inflammatory elicitor lipopolysaccharide lead to a marked increase of IDO1, which was strongly related to emotional deficits (Heisler and O’Connor, 2015). In line with the previous study, our results found that chronic drinking increased mRNA and protein expressions of IDO1 in the hippocampus, cerebral cortex, and amygdala. Different from IDO1, the expression of IDO2 was observed only at mRNA level in the hippocampus and amygdala, and the expression of TDO could not be detected in all these brain regions either at mRNA level or at protein level. In fact, IDO2 is a recently discovered homolog of IDO and shows much lower enzymatic activity as compared with IDO1 (Fatokun et al., 2013). Previous studies demonstrated that proinflammatory stimuli only increased or had no impact on IDO2 mRNA levels (Ball et al., 2007; Divanovic et al., 2012). Therefore, IDO1 is highly associated with the development of neurological damage after chronic ethanol addiction/withdrawal. We do not exclude, however, the possibility that IDO2 and TDO may be highly expressed in other brain regions and exert important functions in behavioral impairment formation in this animal model. Related studies also pointed out that different IDO subtypes may participate in different types of depression (Qin et al., 2018).

A recent study found that the IDO/KYN pathway mediated the effects of proinflammatory cytokines on the brain (Dantzer et al., 2011). In order to confirm whether the KYN/TRP pathway was involved in drinking-induced emotional and cognitive impairments, IDO1 inhibitor 1-MT was administrated from the 4th to 8th week. In our results, depressive- and anxiety-like behaviors were accompanied by the upregulation of IDO1 expression. For depressive-like behavior, a significant increase in immobility time was observed in both the forced swimming test and tail suspension test, which had been widely used to study animals with depression. These helpless behaviors were similar to those of individuals with major depressive disorder (Xu et al., 2006, 2012). For anxiety behavior, less time spent in the open arms and a decreased number of open arm entries were observed in mice exposed to alcohol in the elevated plus maze test. The behavioral deficit of model mice was also found in the marble-burying test. All these results were consistent with previous studies (Gong et al., 2017; Macedo et al., 2018). Interestingly, chronic 1-MT treatment reversed drinking-induced depressive-like and anxiety-like behaviors. Memory deficit is also the common symptoms in ethanol addiction/withdrawal individuals (Topiwala and Ebmeier, 2018). Four weeks of ethanol drinking induced obvious memory impairment, which was demonstrated by the longer latency to find the platform and fewer mice to cross platform. Chronic 1-MT treatment ameliorated drinking-induced memory impairment. The above results indicated that the development of neurological damage was mediated by IDO1. Psychostimulants/psycholeptics have been shown to induce the stimulation/inhibition of the central nervous system (CNS), causing enhancement/weakness to motor function and affecting behavioral performance (Singh et al., 2011). To investigate whether efficacy of 1-MT on emotional deficits was related to CNS stimulation/inhibition, each mouse received a locomotor activity test, an index of wakefulness or alertness of mental activity (Patil et al., 2015). No difference of locomotor activity was observed among groups, indicating that neither 1-MT nor drinking interfered with the accuracy of the results obtained from behavioral tests.

**FIGURE 10:**
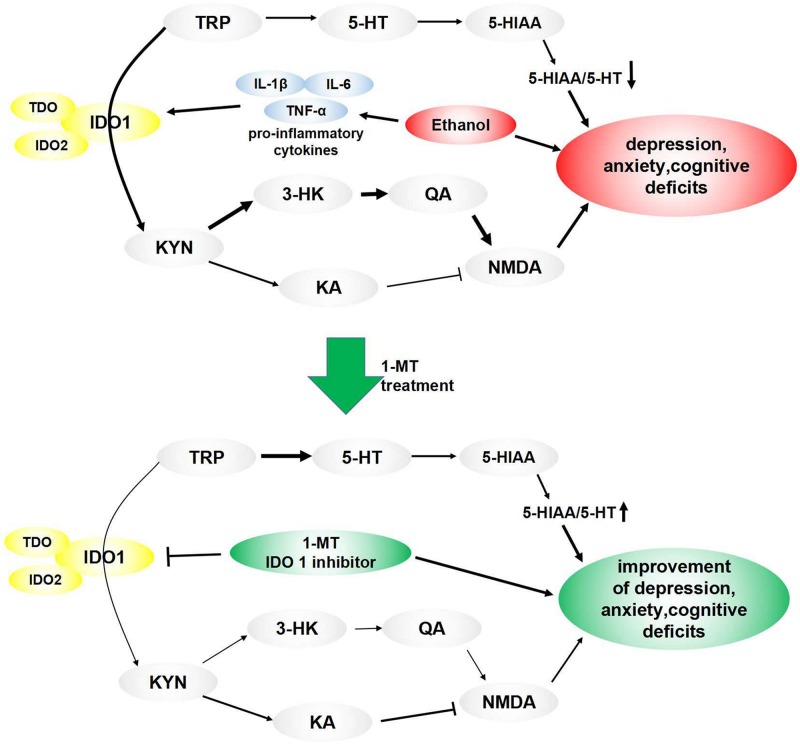
Pathways linking IDO-mediated KYN/TRP mechanism to behavioral changes: ethanol induced overexpression of proinflammatory cytokines, which activated IDO, especially IDO1. TRP’s metabolic pathway was altered when IDO1 was activated, causing a decrease of 5-HT, which induced a decrease of the 5-HT/TRP ratio and 5-HIAA/5-HT ratio, ultimately leading to depressive- and anxiety-like behaviors. Meanwhile, most of the TRP was metabolized into KYN when IDO1 was activated, which broke the balance of KYN’s metabolic pathway. In the normal state, KYN’s metabolite is KA, which is an NMDA antagonist and is beneficial to memory function. When IDO1 was activated, most of KYN convert to QA, which is an NMDA receptor agonist and is a key contributor to increased neurotoxicity and cognitive deficits. These imbalances and behavioral deficits induced by drinking were improved by treatment of IDO1 inhibitor 1-MT.

5-hydroxytryptamine and KYN are two major metabolites of TRP produced under the condition of activation of metabolic enzymes, including IDO (O’Connor et al., 2009b). In normal nerve cells, most of the TRP is metabolized to 5-HT, while a small proportion are converted to KYN. When IDO1 is activated by neuroinflammation, less TRP is metabolized to 5-HT, leading to a deficiency of 5-HT (O’Connor et al., 2009a), a neurotransmitter mediating the process of produce feelings of calmness, relaxation, and contentment. According to the monoamine hypothesis of depression and anxiety, when the amount of 5-HT is reduced, depleted, or dysfunctional for some reason, these disorders will ensue (Badawy, 2013). IDO can be regarded as a pivot point for the regulation of immune processes and monoaminergic systems. Lower TRP levels and abnormal IDO expression were found in the depression and anxiety animal model (Kim et al., 2012). Curiously, the increased KYN/TRP ratio, decreased 5-HT/TRP ratio, and decreased 5-HT/5-HIAA ratio were observed in our study, indicating the balance of TRP metabolites was shifted from 5-HT synthesis to KYN formation in the brain of mice exposed to alcohol. Moreover, 1-MT treatment reversed this imbalance of TRP metabolites and increased the 5-HT/5-HIAA ratio. Parallel to 5-HT, KYN and its metabolites were also related to neuroinflammation-induced brain damage (Overstreet, 2012; Parrott and O’Connor, 2015). KYN is metabolized through two main pathways (Myint et al., 2007). In the major rate-limiting pathway, KYN produces 3-HK, which is then metabolized to the N-methyl-D-aspartate (NMDA) receptor agonist QA, which is a potentially neurotoxic compound. In another pathway, KYN produces KA, an NMDA antagonist thought to have a neuroprotective function (Schwarcz et al., 1983). Under normal conditions, KYN is converted to KA. In the presence of neuroinflammation, most of KYN is metabolized to QA, causing a series of neurotoxic reactions (Sublette and Postolache, 2012). In the present study, an increase in the 3-HK/KA ratio and high levels of QA were observed in the brain of model mice. Dominance production of QA over KA induced NMDA receptor activation, which can lead to an increase of glutamatergic activity and neurotoxicity, ultimately causing memory impairment or mood disorders (Jiang et al., 2018). These abnormal expressions were normalized by chronic 1-MT treatment. The above results demonstrated the participation of the IDO1-mediated KYN/TRP pathway in the formation of drinking-induced emotional deficits in mice. Based on our results, we have drawn a schematic illustration (Figure 10) to explain the intracellular mechanism of the KYN/TRP pathway in mediating the process of emotional deficits in response to chronic drinking.

Despite the interesting findings, our study still contains limitations. First, the same group of mice went through several behavioral tests in 1 day, in which circumstance the animal behaviors may have been affected by the previous experience and body condition. During the experiments, we first carried out behavior tests that required less energy and then performed tests that required a lot of energy, and animals were allowed a rest between the two behavior tests, but the results of behavioral experiments may still have been affected by energy consumption and other possible factors that could not be denied. The experiment should be designed to perform the stressful assays on separate groups of mice. Second, the finding obtained from our study that IDO1 was involved in the development of emotional and memory deficits and mediated the function of KYN/TRP pathway was based solely on the pharmacological inhibition of IDO using 1-MT. However, this drug may have off-target effects and pharmacokinetic issues. The specific role of IDO1 in behavioral deficits induced by ethanol drinking should be confirmed by using other antagonists and agonists, as well as the genetic knockdown and transgenic overexpression of IDO1. These defects in the present study should be remedied in our further research to avoid any inaccuracy in the results.

## Conclusion

In summary, our study demonstrated that drinking-induced behavioral impairment was associated with overexpression of proinflammatory cytokines in the brain, and the IDO1-mediated KYN/TRP pathway in the hippocampus, cerebral cortex, and amygdala plays a pivotal role in the development of behavioral deficits in mice exposed to alcohol.

## Data Availability Statement

The datasets generated for this study are available on request to the corresponding author.

## Ethics Statement

The animal study was reviewed and approved by The Animal Management and Ethics Committee Wenzhou Medical University.

## Author Contributions

XJ, QL, and XY designed the research study. XJ, LC, QY, and XY conducted the experiments. ZC and LX analyzed the data. QL and XY wrote the manuscript.

## Conflict of Interest

The authors declare that the research was conducted in the absence of any commercial or financial relationships that could be construed as a potential conflict of interest.

## Supplementary Material

FIGURE S1 Protein expressions of TDO in the hippocampus, cerebral cortex and amygdala in drinking mice at 0, 2, and 4 weeks. *n* = 8 per group. Con, control group, mod, model group (drinking group).

FIGURE S2 Effects of IDO inhibitor 1-MT (0.1, 1 mg/kg) on locomotor activity. *n* = 8 per group.

FIGURE S3 Effects of IDO inhibitor 1-MT (0.1, 1 mg/kg) on IDO1 expressions in the hippocampus, cerebral cortex and amygdala of mice. *n* = 8 per group, and data were assessed by multi-way ANOVA followed by a Duncan test. ^∗^*p* < 0.05, ^∗∗^*p* < 0.01 and ^∗∗∗^*p* < 0.001 compared with control group; #*p* < 0.05 compared with drinking group.

FIGURE S4 Effects of 1-MT (0.1, 1 mg/kg) on KYN/TRP ratio **(A–C)** and 5-HT/TRP ratio **(D–F)** in the hippocampus, cerebral cortex, and amygdala in drinking mice. *n* = 8 per group, data were assessed by multi-way ANOVA followed by a Duncan test. ^∗^*p* < 0.05, ^∗∗^*p* < 0.01, and ^∗∗∗^*p* < 0.001 compared with control group; #*p* < 0.05 compared with drinking group.

FIGURE S5 Effects of 1-MT (0.1, 1 mg/kg) on 5-HIAA/5-HT ratio **(A–C)**, 3-HK/KA ratio **(D–F)** and QA level **(G–I)** in the hippocampus, cerebral cortex, and amygdala of drinking mice. *n* = 8 per group, and data were assessed by multi-way ANOVA followed by Duncan test. ^∗^*p* < 0.05, ^∗∗^*p* < 0.01, and ^∗∗∗^*p* < 0.001 compared with control group; #*p* < 0.05 compared with drinking group.

FIGURE S6 Effects of 1-MT on locomotor activity **(A)**, ethanol preference test **(B)**, forced swimming test **(C)**, tail suspension test **(D)**, marble-burying test **(E)**, and elevated plus maze test **(F–H)** in control mice. *n* = 6 per group.

FIGURE S7 Effects of 1-MT on IDO1 expressions in hippocampus **(A)**, cerebral cortex **(B)**, and amygdala **(C)** of control mice. *n* = 6 per group. Con, control group.
