# Supplementary figures and images for: Indoleamine-2,3-Dioxygenase Mediates Emotional Deficits by the Kynurenine/Tryptophan Pathway in the Ethanol Addiction/Withdrawal Mouse Model
Source: Front Cell Neurosci. 2020 Feb 11;14:11. doi: 10.3389/fncel.2020.00011 (PMC7026684; doi:10.3389/fncel.2020.00011)

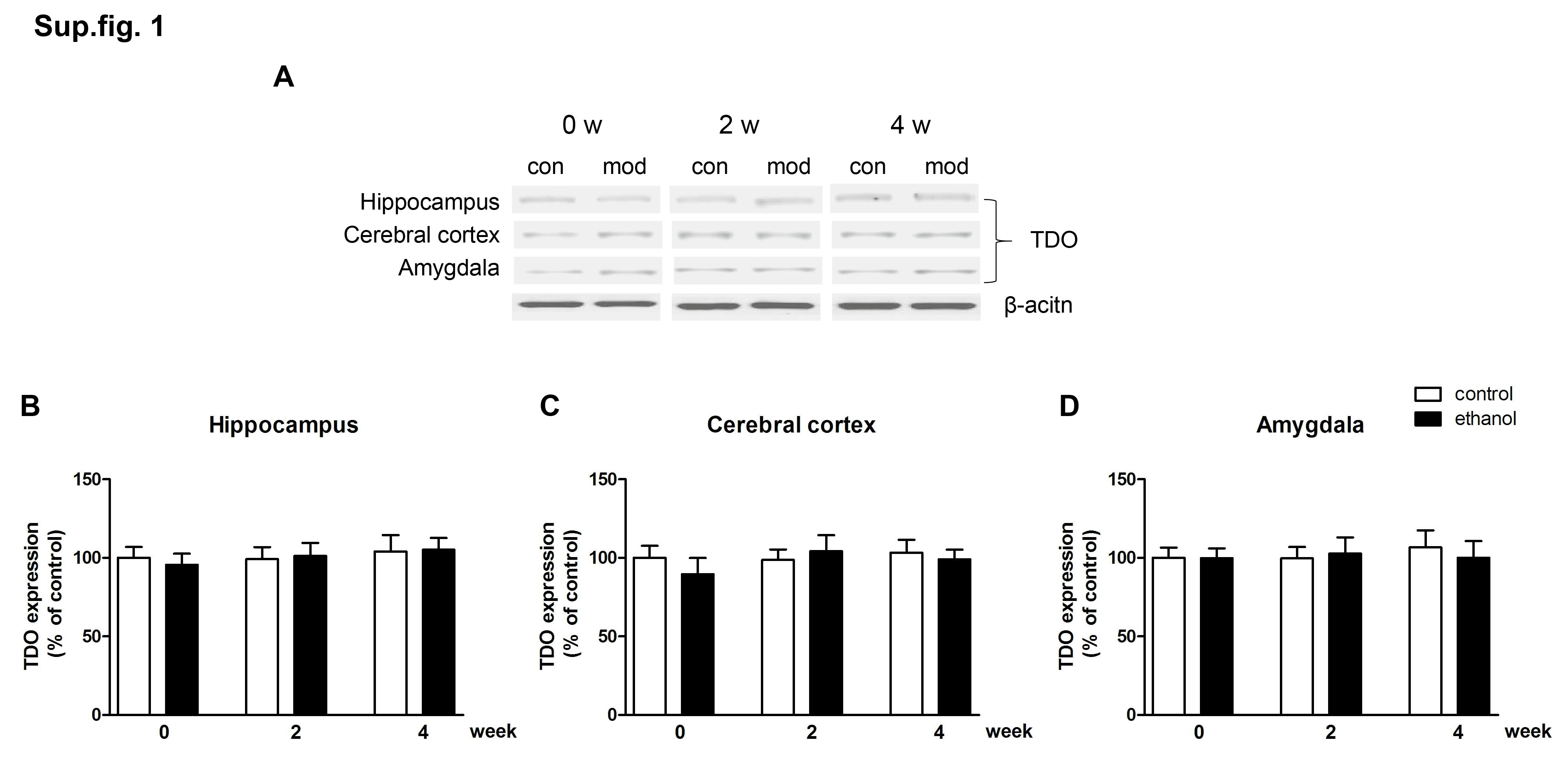

Supplement: FIGURE S1 — Protein expressions of TDO in the hippocampus, cerebral cortex and amygdala in drinking mice at 0, 2, and 4 weeks. n = 8 per group. Con, control group, mod, model group (drinking group). [file Image_1.JPEG]

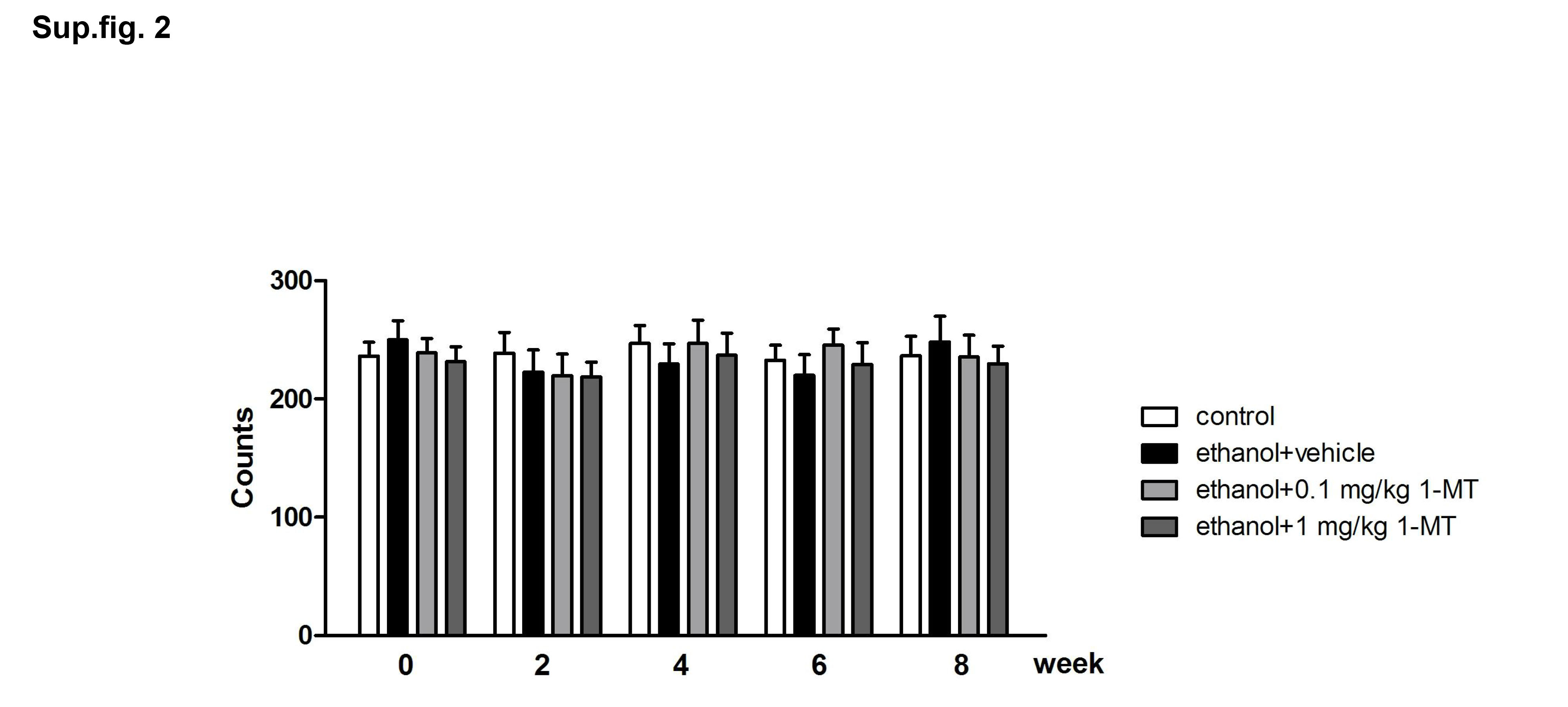

Supplement: FIGURE S2 — Effects of IDO inhibitor 1-MT (0.1, 1 mg/kg) on locomotor activity. n = 8 per group. [file Image_2.JPEG]

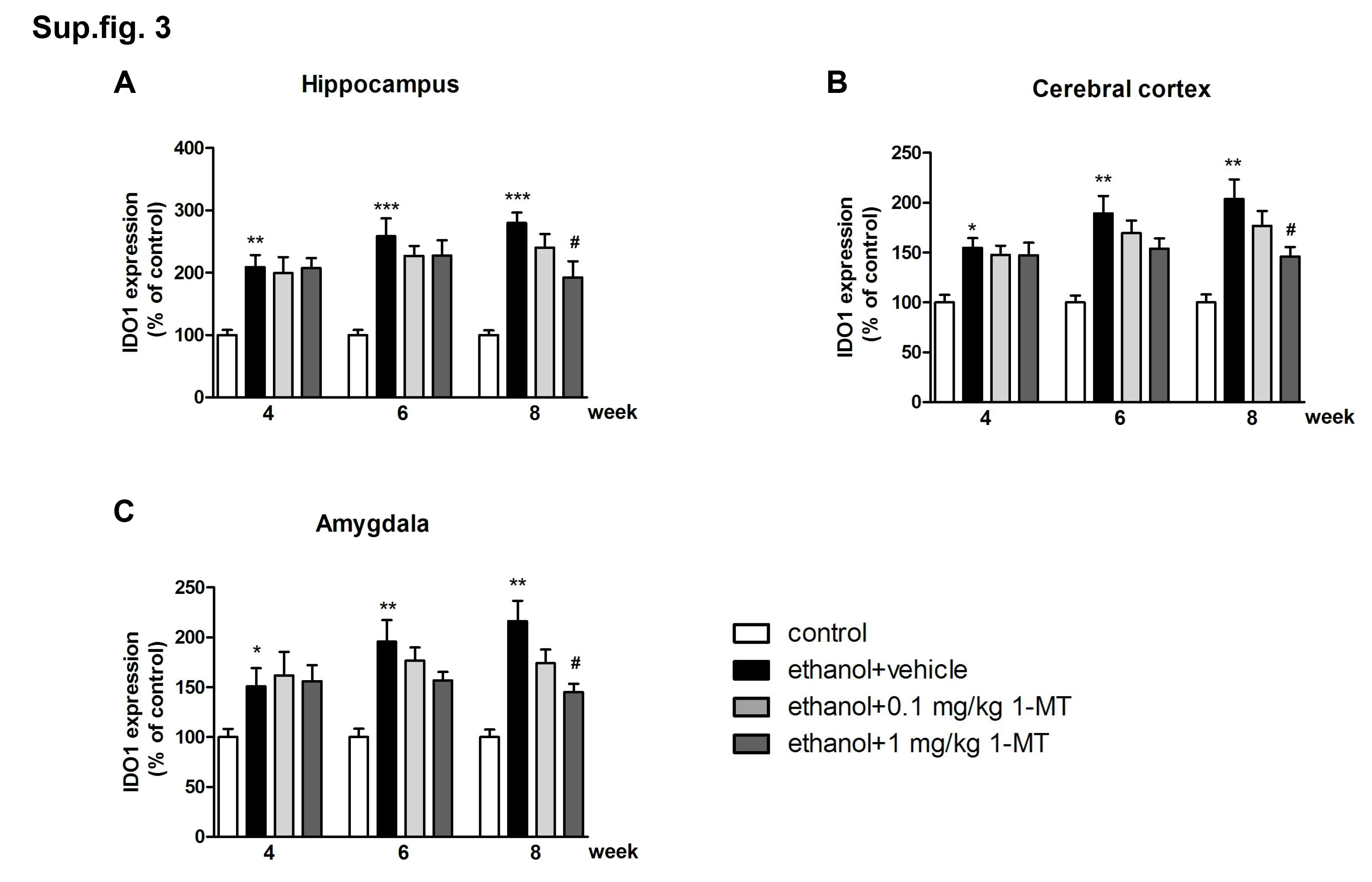

Supplement: FIGURE S3 — Effects of IDO inhibitor 1-MT (0.1, 1 mg/kg) on IDO1 expressions in the hippocampus, cerebral cortex and amygdala of mice. n = 8 per group, and data were assessed by multi-way ANOVA followed by a Duncan test. ∗p < 0.05, ∗∗p < 0.01 and ∗∗∗p < 0.001 compared with control group; #p < 0.05 compared with drinking group. [file Image_3.JPEG]

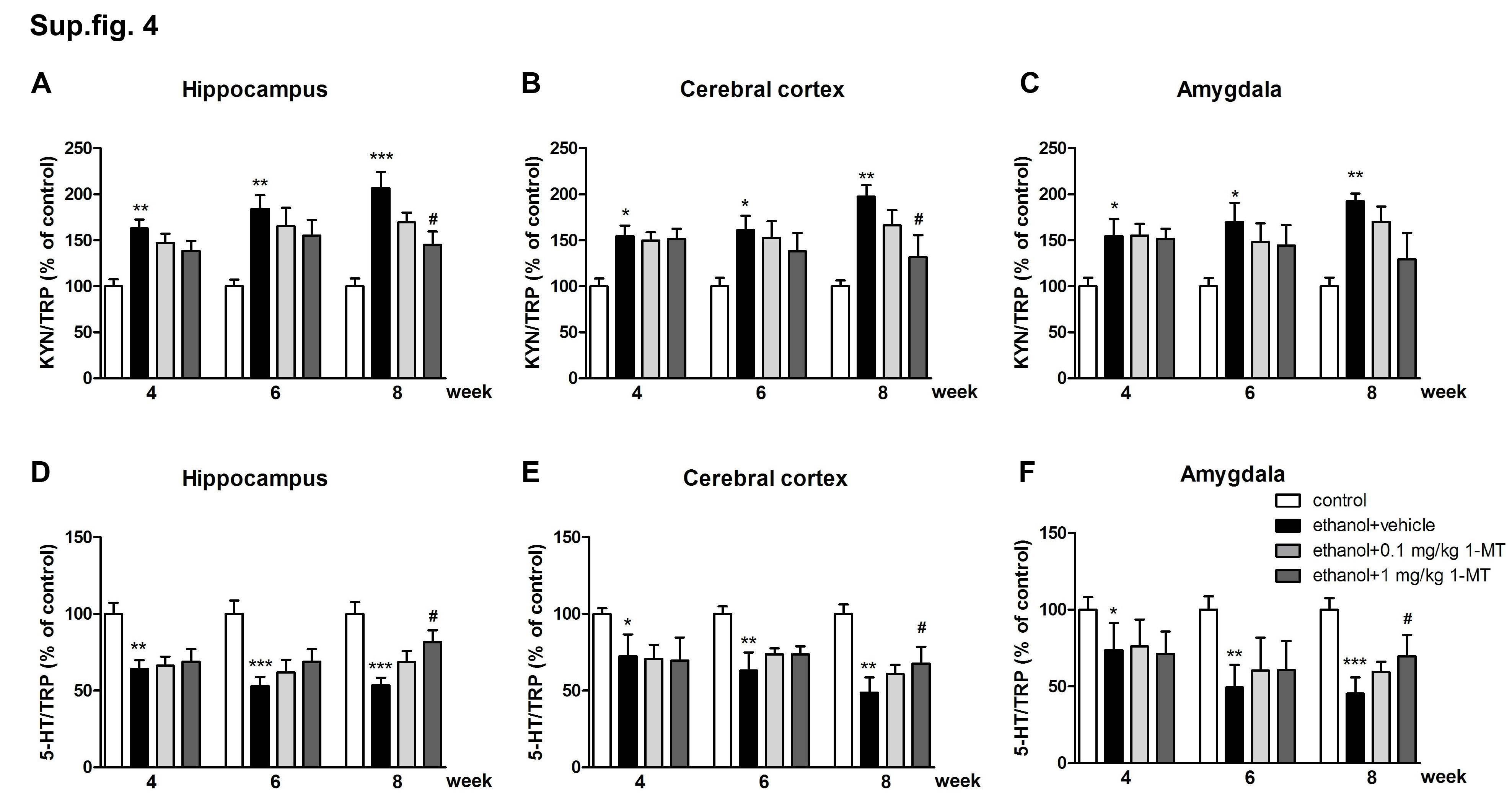

Supplement: FIGURE S4 — Effects of 1-MT (0.1, 1 mg/kg) on KYN/TRP ratio (A–C) and 5-HT/TRP ratio (D–F) in the hippocampus, cerebral cortex, and amygdala in drinking mice. n = 8 per group, data were assessed by multi-way ANOVA followed by a Duncan test. ∗p < 0.05, ∗∗p < 0.01, and ∗∗∗p < 0.001 compared with control group; #p < 0.05 compared with drinking group. [file Image_4.JPEG]

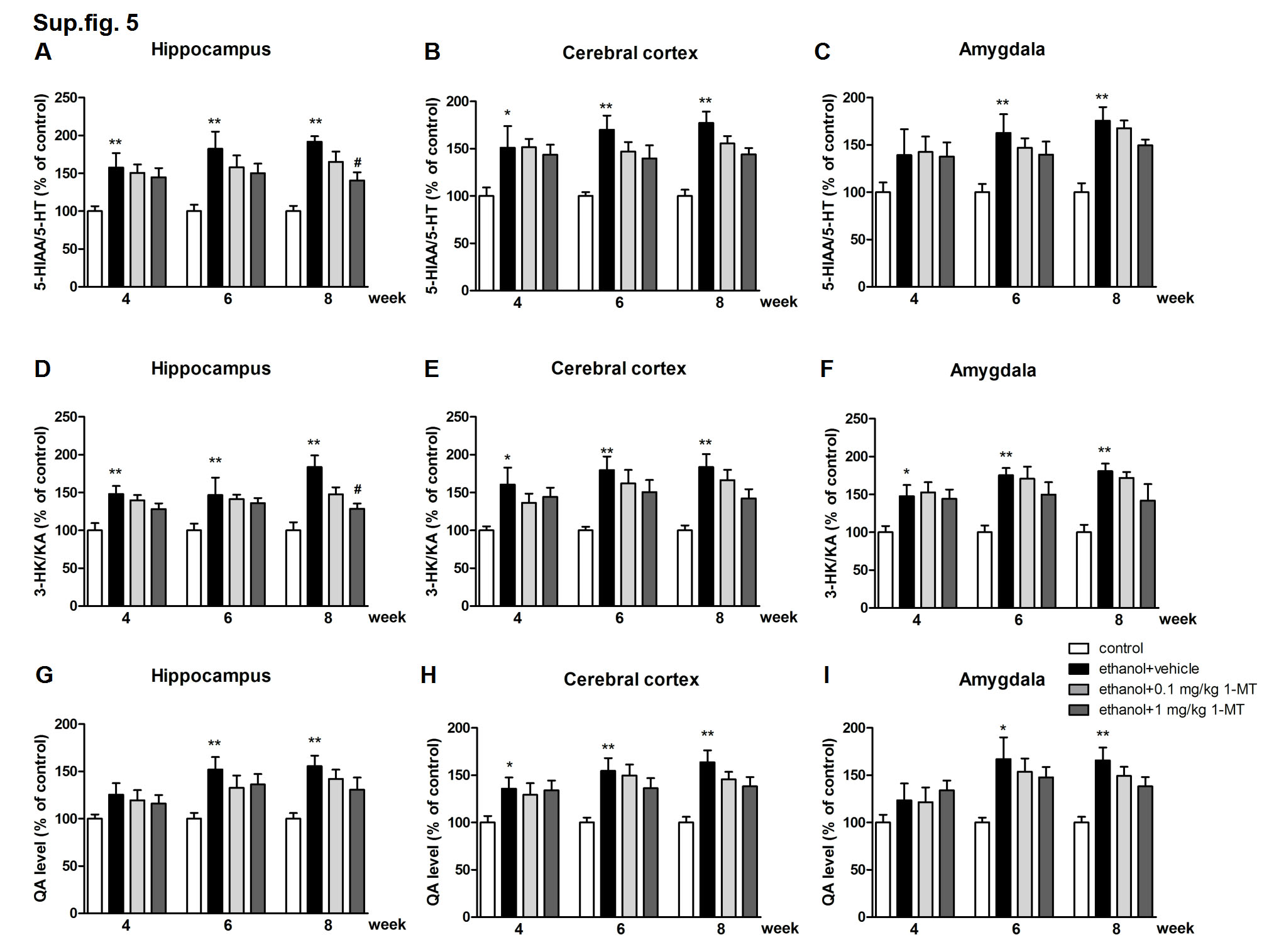

Supplement: FIGURE S5 — Effects of 1-MT (0.1, 1 mg/kg) on 5-HIAA/5-HT ratio (A–C), 3-HK/KA ratio (D–F) and QA level (G–I) in the hippocampus, cerebral cortex, and amygdala of drinking mice. n = 8 per group, and data were assessed by multi-way ANOVA followed by Duncan test. ∗p < 0.05, ∗∗p < 0.01, and ∗∗∗p < 0.001 compared with control group; #p < 0.05 compared with drinking group. [file Image_5.JPEG]

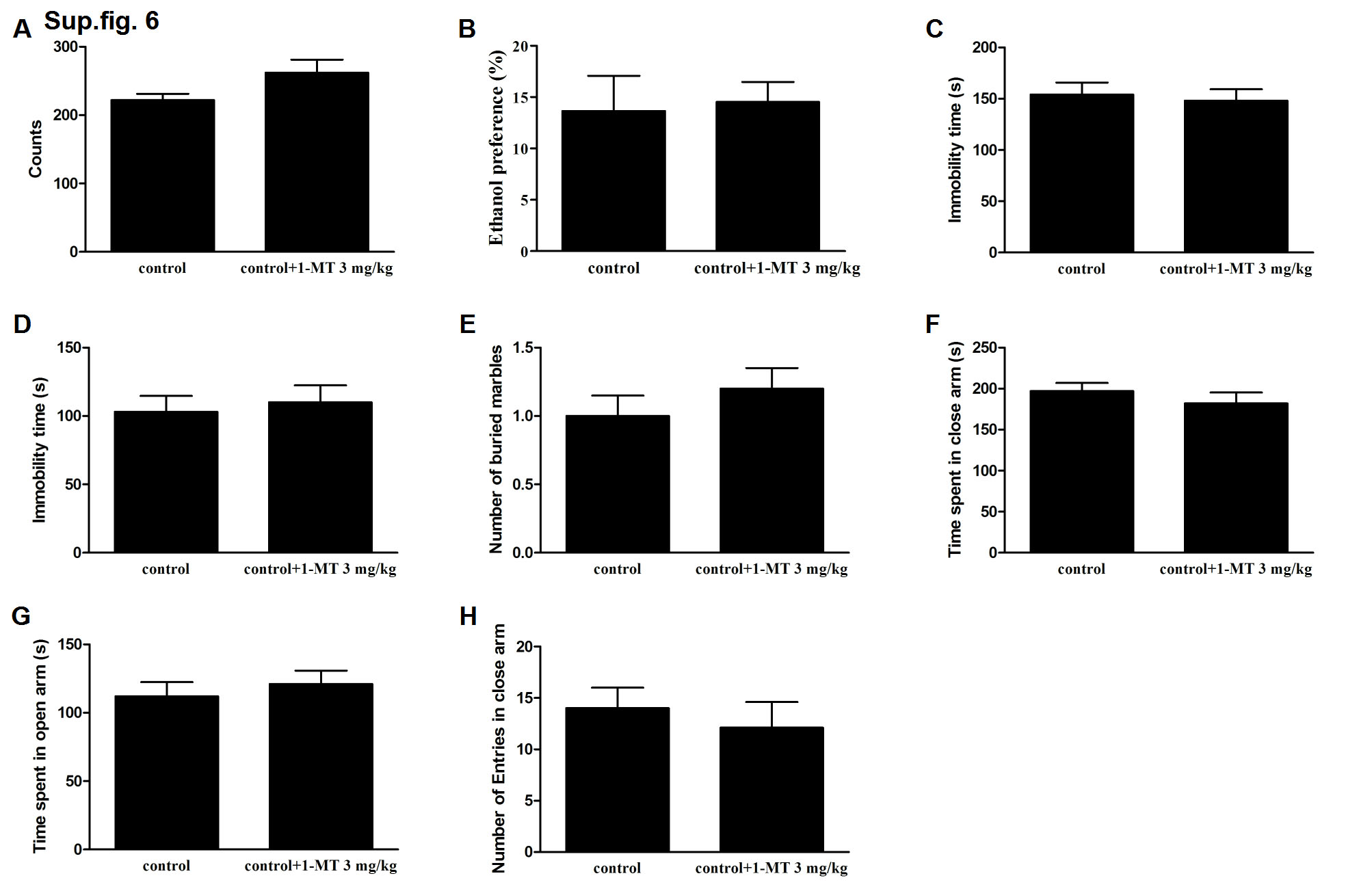

Supplement: FIGURE S6 — Effects of 1-MT on locomotor activity (A), ethanol preference test (B), forced swimming test (C), tail suspension test (D), marble-burying test (E), and elevated plus maze test (F–H) in control mice. n = 6 per group. [file Image_6.JPEG]

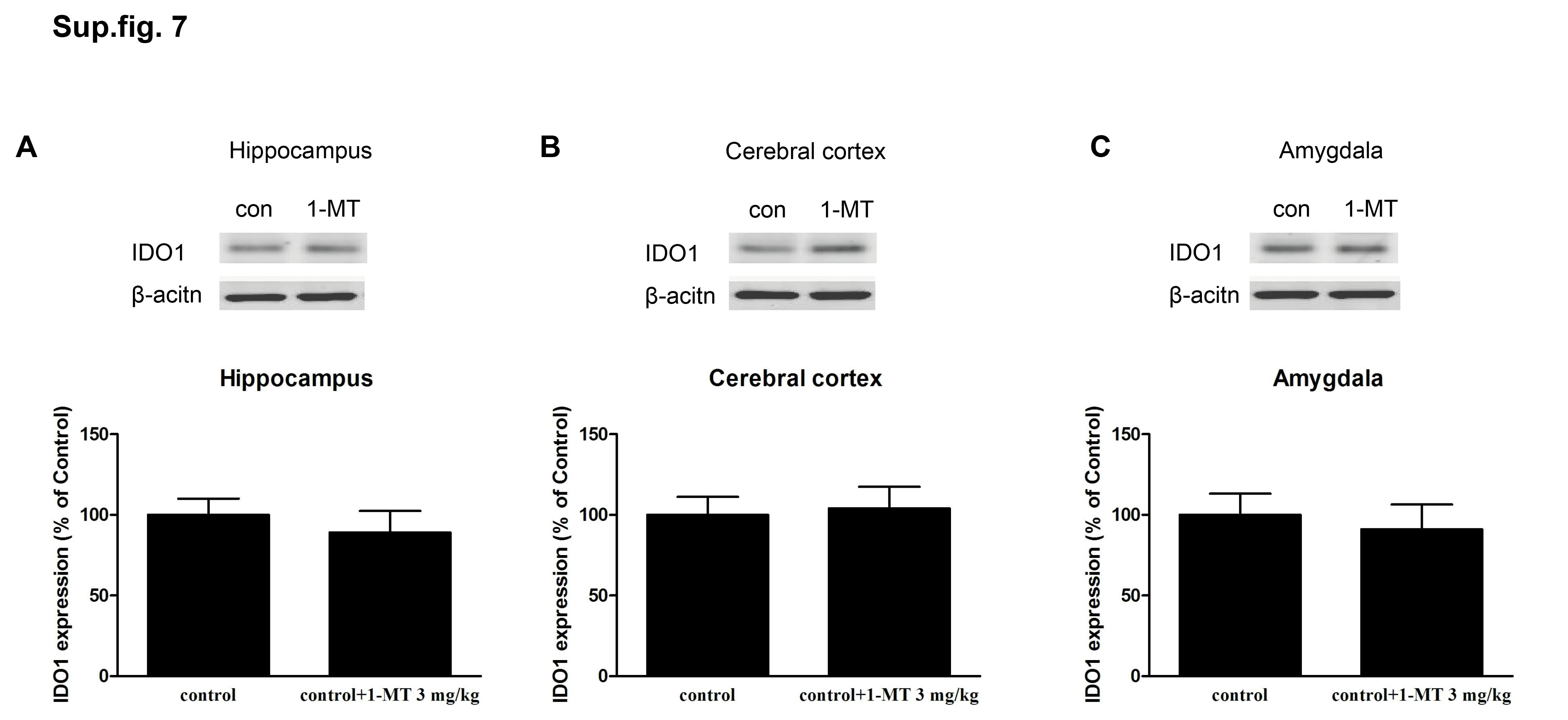

Supplement: FIGURE S7 — Effects of 1-MT on IDO1 expressions in hippocampus (A), cerebral cortex (B), and amygdala (C) of control mice. n = 6 per group. Con, control group. [file Image_7.JPEG]
