# Supplementary material for: Indoleamine-2,3-Dioxygenase Mediates Emotional Deficits by the Kynurenine/Tryptophan Pathway in the Ethanol Addiction/Withdrawal Mouse Model
Source: Front Cell Neurosci. 2020 Feb 11;14:11. doi: 10.3389/fncel.2020.00011 (PMC7026684; doi:10.3389/fncel.2020.00011)
Supplement: Supplementary file 8 [file Data_Sheet_1.PDF]

Table S1 Neuroinflammation increases flux through the kynurenine pathway of tryptophan metabolism at 4th week

Metabolite	Hippocampus (μM)			Cerebral cortex (μM)			Amygdala (μM)		
	Saline	model	Model+1-MT	Saline	model	Model+1-MT	Saline	model	Model+1-MT
TRY	91.9±7.8	92.9±10.5	102.6±11.8	82.5±8.9	78.1±14.5	81.6±13.8	73.4±8.5	78.6±14.7	74.9±12.7
KYN	0.81±0.07	1.49±0.15**	1.28±0.18$^{\#}$	0.77±0.07	1.25±0.14*	1.19±0.08	0.57±0.12	0.84±0.19**	0.78±0.16
5-HT	95.7±12.4	59.7±16.5**	77.1±13.6	104.8±12.7	59.9±15.4**	65.7±14.4	76.4±7.2	46.7±6.8*	48.3±6.2
5-HIAA	52.2±6.4	56.3±7.5	55.5±7.9	42.7±5.8	39.3±9.2	42.6±5.9	27.2±2.2	29.1±1.9	28.6±3.1
3-HK	0.15±0.03	0.22±0.05*	0.21±0.07	0.23±0.05	0.30±0.03	0.28±0.05	0.19±0.02	0.28±0.05*	0.26±0.06
KA	0.27±0.03	0.23±0.06	0.23±0.05	0.31±0.07	0.23±0.06	0.23±0.07	0.25±0.04	0.23±0.03	0.27±0.04

Table values are expressed as mean ± SD, n=8. *$p<0.05$ and **$p<0.01$ compared with control group; # $p < 0.05$ compared with model drinking group. Tryptophan: TRY, Kynurenine: KYN, 5-hydroxytryptamine: 5-HT, 5-hydroxyindole acetic acid: 5-HIAA, 3-Hydroxykynurenine: 3-HK), Kynurenic acid: KA.

Table S2 Neuroinflammation increases flux through the kynurenine pathway of tryptophan metabolism at 6th week

Metabolite	Hippocampus (μM)			Cerebral cortex (μM)			Amygdala (μM)		
	Saline	model	Model+1-MT	Saline	model	Model+1-MT	Saline	model	Model+1-MT
TRY	92.4±8.3	95.5±13.1	101.5±15.6	82.7±9.4	80.2±131	87.5±16.4	72.5±6.4	78.9±13.2	73.1±14.3
KYN	0.83±0.08	1.54±0.14**	1.29±0.13#	0.77±0.06	1.32±0.14**	1.08±0.14	0.56±0.13	0.86±0.19**	0.81±0.17
5-HT	94.1±9.5	50.7±15.6***	79.7±18.4#	105.8±10.3	52.1±10.7**	67.7±9.4	73.1±6.8	38.2±4.9**	52.4±4.4#
5-HIAA	53.1±7.2	53.6±6.5	62.0±7.4	43.0±8.5	39.6±9.2	39.5±7.4	24.1±1.7	27.2±1.8	29.3±2.0
3-HK	0.14±0.04	0.24±0.04*	0.18±0.05	0.21±0.08	0.32±0.07*	0.27±0.06	0.18±0.04	0.31±0.04**	0.24±0.04
KA	0.27±0.03	0.19±0.04	0.20±0.06	0.30±0.05	0.20±0.08	0.24±0.06	0.24±0.04	0.20±0.04	0.24±0.05

Table values are expressed as mean ± SD, n=8. *p<0.05, **p<0.01 and ***p<0.001 compared with control group; # p < 0.05 compared with model drinking group. Tryptophan: TRY, Kynurenine: KYN, 5-hydroxytryptamine: 5-HT, 5-hydroxyindole acetic acid: 5-HIAA, 3-Hydroxykynurenine: 3-HK), Kynurenic acid: KA.

Table S3 Neuroinflammation increases flux through the kynurenine pathway of tryptophan metabolism at 8th week

Metabolite	Hippocampus (μM)			Cerebral cortex (μM)			Amygdala (μM)		
	Saline	model	Model+1-MT	Saline	model	Model+1-MT	Saline	model	Model+1-MT
TRY	92.5±10.1	95.3±10.5	103.8±16.2	81.4±8.5	86.4±12.4	87.6±13.6	72.5±6.9	72.5±11.2	68.7±14.2
KYN	0.85±0.08	1.85±0.16***	1.1±0.17##	0.75±0.06	1.52±0.15***	0.81±0.16###	0.54±0.08	1.2±0.17***	0.78±0.16##
5-HT	96.3±8.5	54.8±14.2***	91.2±9.7###	107.1±7.2	43.2±8.6***	90.4±10.6###	76.1±3.5	35.3±5.2**	72.0±6.9##
5-HIAA	51.3±8.2	54.4±8.7	56.9±7.6	42.8±7.4	43.5±6.2	44.0±8.4	26.8±1.9	25.1±2.3	23.3±2.0
3-HK	0.13±0.02	0.25±0.06*	0.16±0.07#	0.23±0.05	0.41±0.08**	0.28±0.05#	0.17±0.02	0.32±0.06**	0.19±0.04#
KA	0.23±0.04	0.15±0.04*	0.21±0.05	0.32±0.07	0.19±0.11*	0.27±0.08#	0.24±0.03	0.19±0.06	0.22±0.03

Table values are expressed as mean ± SD, n=8. *p<0.05, **p<0.01 and ***p<0.001 compared with control group; # p < 0.05, ## p < 0.01 and ### p < 0.001 compared with model drinking group. Tryptophan: TRY, Kynurenine: KYN, 5-hydroxytryptamine: 5-HT, 5-hydroxyindole acetic acid: 5-HIAA, 3-Hydroxykynurenine: 3-HK), Kynurenic acid: KA.

Table S4 Neuroinflammation increases flux through the kynurenine pathway of tryptophan metabolism in mice of control and control+1-MT 3mg/kg group

Metabolite	Hippocampus (μM)		Cerebral cortex (μM)		Amygdala (μM)	
	control	control+1-MT	control	control+1-MT	control	control+1-MT
TRY	88.6±8.5	91.4±9.3	72.7±7.7	74.9±8.5	74.3±9.6	76.8±8.9
KYN	0.82±0.06	0.86±0.09	0.56±0.1	0.58±0.08	0.59±0.1	0.58±0.11
5-HT	93.2±11.2	92.4±10.4	74.9±6.8	75.4.7±7.2	75.8±8.1	76.7±7.5
5-HIAA	51.6±5.7	53.2±7.2	29.1±2.5	28.6±2.1	28.4±2.4	27.9±2.1
3-HK	0.16±0.04	0.14±0.05	0.21±0.04	0.18±0.04	0.2±0.05	0.21±0.06
KA	0.25±0.04	0.24±0.05	0.24±0.05	0.23±0.04	0.24±0.02	0.23±0.06
QA	0.47±0.05	0.48±0.07	0.39±0.07	0.41±0.05	0.29±0.01	0.31±0.02

Table values are expressed as mean ± SD, n=6. Tryptophan: TRY, Kynurenine: KYN, 5-hydroxytryptamine: 5-HT, 5-hydroxyindole acetic acid: 5-HIAA, 3-Hydroxykynurenine: 3-HK), Kynurenic acid: KA, Quinolinic acid: QA.
